# Automatic Detection of Atrial Fibrillation Based on CNN-LSTM and Shortcut Connection

**DOI:** 10.3390/healthcare8020139

**Published:** 2020-05-20

**Authors:** Yongjie Ping, Chao Chen, Lu Wu, Yinglong Wang, Minglei Shu

**Affiliations:** 1 College of Computer Science and Engineering, Shandong University of Science and Technology, Qingdao 266590, China; pyjboxmail@163.com (Y.P.); lwu@qlu.edu.cn (L.W.); 2 Shandong Artificial Intelligence Institute, Shandong Computer Science Center (National Supercomputer Center in Jinan), Qilu University of Technology (Shandong Academy of Sciences), Jinan 250014, China; chench@sdas.org (C.C.); wangylscsc@126.com (Y.W.)

**Keywords:** atrial fibrillation (AF), long short-term memory (LSTM), CNN with shortcut connection

## Abstract

Atrial fibrillation (AF) is one of the most common persistent arrhythmias, which has a close connection to a large number of cardiovascular diseases. However, if spotted early, the diagnosis of AF can improve the effectiveness of clinical treatment and effectively prevent serious complications. In this paper, a combination of an 8-layer convolutional neural network (CNN) with a shortcut connection and 1-layer long short-term memory (LSTM), named 8CSL, was proposed for the Electrocardiogram (ECG) classification task. Compared with recurrent neural networks (RNN) and multi-scale convolution neural networks (MCNN), not only can 8CSL extract features skillfully, but also deal with long-term dependency between data. In particular, 8CSL includes eight shortcut connections that can improve the speed of the data transmission and processing as a result of the shortcut connections. The model was evaluated on the base of the test set of the Computing in Cardiology Challenge 2017 dataset with the F1 score. The ECG recordings were cropped or padded to the same length. After 10-fold cross-validation, the average test F1 score was 84.89%, 89.55%, and 85.64% when the segment length was 5, 10, 20 s, respectively. The experiment results demonstrate excellent performance with potential practical applications.

## 1. Introduction

Cardiovascular disease is one of the major causes of death worldwide. According to an uncompleted statistic, three million people die of cardiovascular disease every year in China (i.e., one patient dies of cardiovascular disease every 10 s 1).

Atrial fibrillation (AF) is one of the most common persistent arrhythmias, which has a close connection to a large number of cardiovascular diseases [1,2,3,4]. When AF occurs, the patient’s heart rate is fast, sometimes up to 100–160 beats/min, and irregular. AF can be subdivided into paroxysmal AF, persistent AF, and permanent AF, according to the duration. However, if detected early, it can improve the clinical treatment effect and effectively prevent the occurrence of serious complications.

The electrocardiogram (ECG), invented by Muirhead in 1872, is a non-invasive method that is widely used in the clinical diagnosis of AF and other types of arrhythmia. Furthermore, ECG records the heartbeats by connecting wires to the wrists. RR interval refers to the time limit between two R waves on an ECG. The normal RR interval should be between 0.6 and 1.0 s. Additionally, AF has distinctive characteristics such as the disappearance of P waves or different RR intervals.

In recent years, researchers have proposed many automatic detection methods before deep learning, and sometimes, the cost of feature extraction is too large to improve the effect significantly. In recent years, with the development of deep learning, convolutional neural networks (CNNs) and recurrent neural networks (RNNs) have been widely used in detecting AF with excellent results. As for CNN, it performs well in feature extraction and achieves good results in image classification and retrieval [5,6,7]. Furthermore, many researchers use CNNs to process and identify AF. For example, Ghiasi et al. [8] proposed a CNN algorithm for automatically detecting AF signals from ECG signals. Pourbabaee et al. [9] developed a new automatic AF detection method based on deep convolutional neural networks (DCNN). Qayyum et al. [10] proposed converting ECG signals into 2D images using short-time Fourier transform and put them into a pre-trained CNN model. Cho et al. [11] proposed an approach for the prediction of AF by using DCNN. Wang et al. [12] adopted the CNN and the improved Elman neural network for detection of AF. Xiong et al. [13] proposed a 16-layer 1D CNN to classify the ECGs including AF with a testing accuracy of 82%. For RNN, it takes the time series of data into account while processing data, so of course, it can also be applied to ECG signals. For example, Schwab et al. [14] introduced a novel task formula to simplify the learning of the time dimension and RNN was used to detect AF signals. Sujadevi et al. [15] explored and adopted three deep learning methods: RNN, the long short term memory network (LSTM), and the gate recurrent unit (GRU) neural network, and achieved accuracies of 0.950, 1.000, and 1.000, respectively, on the MIT-BIH Physionet dataset. Faust et al. [16] proposed the LSTM combined with RR intervals to detect AF. Additionally, studies have shown that LSTM is superior to other traditional RNN architectures [17]. Wang et al. [18] developed a novel approach of an 11-layer neural network and the modified Elman neural network (MENN) for the automated AF detection, and the proposed model achieved exceptional results with the accuracy, sensitivity, and specificity of 97.4%, 97.9%, and 97.1%, respectively. Jonathan et al. [19] combined a signal quality index (SQI) algorithm and CNN to detect AF, the results achieved on the test dataset were an overall F1 score of 0.82. In the above research, the researchers did not better solve the long-term dependence between ECG data under the premise of ensuring accurate feature extraction, and did not pay attention to the time of processing and transmitting data. Shortcut connection has also been proven in theory and practice for a long time. In [20,21,22], the importance of shortcut connections in improving neural networks was introduced through theoretical research. In [23,24], some intermediate layers were connected directly with auxiliary classifiers for addressing vanishing and exploding gradients. In [25,26], to solve the problems of response, gradient, and propagation error in the middle layer, the validity of the shortcut connection was verified through a comparison of several common methods and the shortcut connection. Table 1 shows the arrangement of the methods and results in the references.

However, some problems still remain. For example, the amount of data that deep learning needs to process is substantial and diverse. Thus, two challenges still exist: (1) the speed of data transmission and processing; and (2) the right deep learning model for the data type to achieve excellent results.

In this paper, we proposed a model that employed CNN based on the shortcut connection and LSTM to address these two challenges. The proposed model was named 8CSL. The main contributions are: (1) The data transmission and the data processing were sped up by 38% by using the shortcut connection; and (2) combining CNN and LSTM, and adjusting the number of network layers and parameters to improve the accuracy of AF detection while ensuring efficient feature extraction where the best F1 score was 89.55%.

The rest of the paper is organized as follows. In Section 2, the basic knowledge of CNN, LSTM, and the shortcut connection is introduced. In Section 3, the Computing in Cardiology Challenge 2017 dataset and the data processing are described. In Section 4, the 8CSL is proposed. In Section 5, the experiments were designed to validate the performance of the proposed model and discuss the effects of different segment lengths.

## 2. Technical Background

### 2.1. Convolutional Neural Network (CNN) Structure

CNNs can extract features skillfully and reduce network complexity at the same time [27]. Weight sharing and receptive field play an important role in this.

#### 2.1.1. Receptive Field

**Figure 1:**
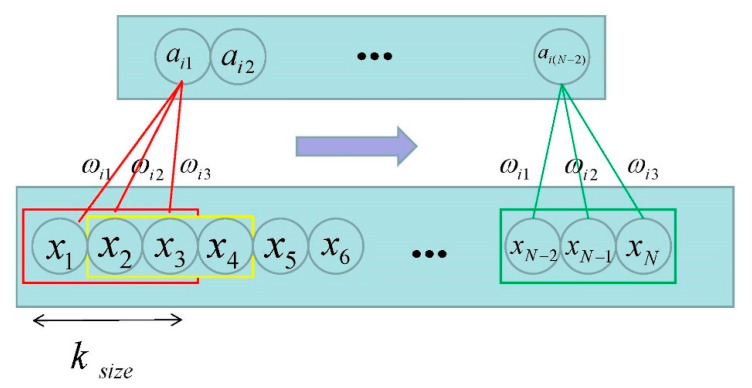
Temporal convolution [28]. (Where N represents the length of the input signal; $k_{size}$ stands for the size of the receptive field; and the three corresponding weights $\left(w_{i1},\;w_{i2},\;\;w_{i3}\right)$ are the ith filter.)

In CNN, each neuron only needs to sense the local part and integrate it at a higher level to obtain global information. The temporal convolution is shown in Figure 1.

#### 2.1.2. Weight Sharing

To further reduce the number of parameters, weight sharing is employed. This means that different convolution kernels working on a CNN will not change the weight of the convolution kernel as the position changes.

### 2.2. Long Short-Term Memory (LSTM) Structure

**Figure 2:**
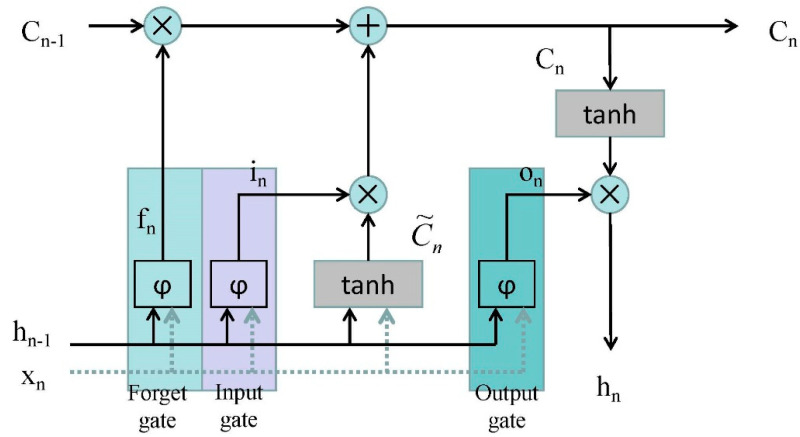
The architecture of a long short-term memory (LSTM) memory block [28]. (Where $X_{n}$ is the input data at time n; $h_{n-1}$ is the data output by LSTM at time n−1; φ is the sigmoid activation function; $i_{n}$ is the input gate; $f_{n}$ is the forget gate; $o_{n}$ is the output gate; and the $C_{n}$ is updated by partially forgetting the existing memory and adding a new content ${\tilde{C}}_{n}$.)

The working mechanism of LSTM is the continuously updated memory $c_{n}$. The LSTM memory block is shown in Figure 2.

The architecture of an LSTM memory block has also been named a cell, which has three gates: the input gate, forget gate, and output gate. Data are sent in the LSTM through the input gate, processed through the sigmoid layer, the status is updated to the cell, and outputted through the output gate. 

It is noteworthy that the output gate depends not only on the input gate and the previous output gate, but also on the current memory [29].

### 2.3. Shortcut Connection

**Figure 3:**
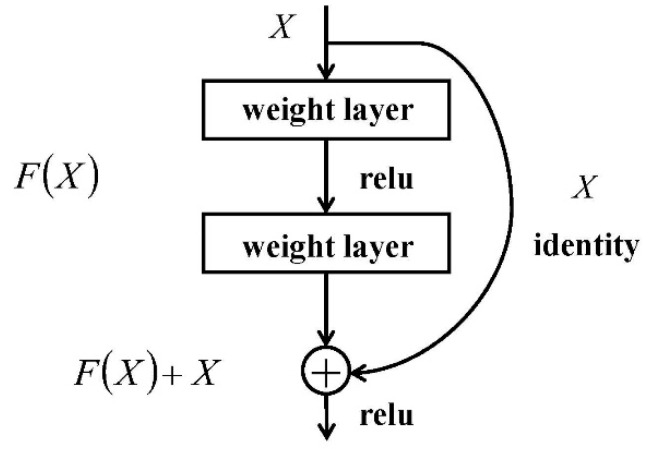
The architecture of a residual block [30]. (Where X is the input data; F(X) is the network map before summation; and F(X)+X is the network map after summation.)

A neural network containing shortcut connections to jump over some layers in residual neural networks is called a residual block. The architecture is shown in Figure 3.

The idea of a shortcut connection of the residual neural network is adopted to streamline the network optimization and speed up data transmission and processing. In this paper, the part of the data were transferred to the shortcut connection and finally pooled with the rest.

## 3. Electrocardiogram (ECG) Data

### 3.1. Data Source

**Figure 4:**
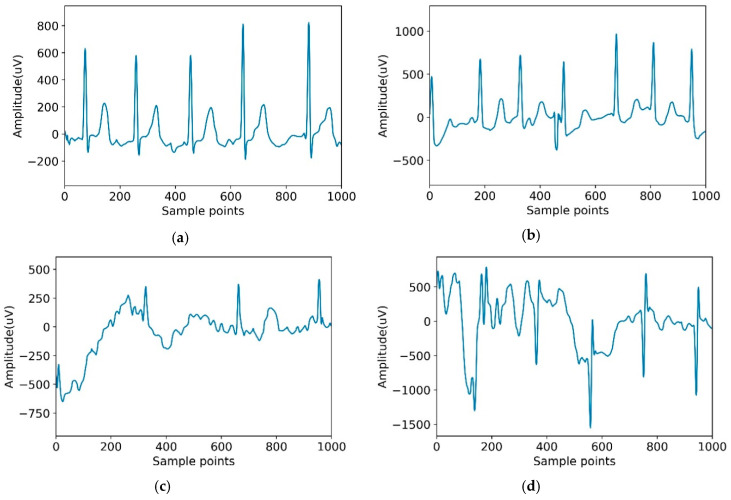
Typical recordings for each of the four classes in the dataset. (**a**) Normal; (**b**) Atrial fibrillation (AF); (**c**) Other; (**d**) Noisy.

The dataset used in this paper was from the Computing in Cardiology Challenge 2017 including 8528 pieces of data ranging in length from 9 s to 61 s. The ECG recordings were collected by the AliveCor device with the data sampling rate of 300 Hz. In this challenge, we treated all non-AF abnormal rhythms as a single class. Datasets were manually annotated as four categories: normal sinus rhythm (N), AF rhythm (A), other rhythms (O), and noisy recordings (∼). Detailed information on the data are shown in Table 2. Figure 4 is an example of four categories.

### 3.2. Data Preprocessing

#### 3.2.1. Normalization

The amplitude of ECG data varies greatly among different people or even the same people with different lead positions [31]. When the data distribution is uniform, the convergence of the neural network is better. Therefore, Equation (1) is used to reduce the impact of different amplitudes in the data: subtract the average value from each ECG data, and then divide it into standard deviations.

(1) $$Normalized\left(X\right)=\frac{X-\bar{X}}{S}$$

where X refers to the ECG recording values, $\bar{X}$ and S refers to the average and standard deviation of these values, correspondingly.

To verify that the normalized data were conducive to the classification of the model, the model was used to classify the normalized data and the unprocessed data, from which the accuracy was obtained. The experimental results are shown in Table 3.

It can be seen from Table 3 that the accuracy of the model processing normalized data was significantly higher than that of the original data.

#### 3.2.2. Data Balance

The distribution of datasets also affects the results of the training. It can be seen from Table 2 that the number of AF and other rhythm ECG data were far less than that of the normal ECG data, and there were only 46 noisy data.

This imbalanced dataset made it more difficult to detect AF than normal ECG. At the same time, because the number of normal ECG was much larger than that of AF, the normal ECG will play a leading role in the process of model training, and the over-fitting phenomenon will appear.

To solve this problem, in this paper, noise and other rhythm ECG data were discarded, and the experiment was converted to detect AF in AF data and normal data. The dataset was randomly divided into a training dataset and test dataset with a proportion of 7:3. To balance the amount of ECG data with normal ECG data, four copies of AF ECG data was added to the training dataset [31].

#### 3.2.3. Cropping

All of the data in the input neural network model must be consistent in length. However, the length of the ECG data was in the range of 9 s to 61 s. Therefore, in this paper, we cropped the data into 5 s segments. The sampling rate of the experimental data was 300 Hz. A total of 1500 points were taken as a segment, and the segment with less than 1500 points was deleted. At the same time, the data were transformed into 10 s and 20 s segments. In this process, longer data should be cropped and shorter data should be deleted. To verify whether the time of data can affect the performance of the model in the experiment, we cropped it into 5 s, 10 s, and 20 s.

## 4. Model

In this paper, three deep learning models were compared: recurrent neural networks (RNN), multi-scale convolution neural networks (MCNN), and the proposed model of the combination of the 8-layer CNN with shortcut connection and one layer LSTM (8CSL) for the ECG classification task.

### 4.1. Recurrent Neural Networks

**Figure 5:**
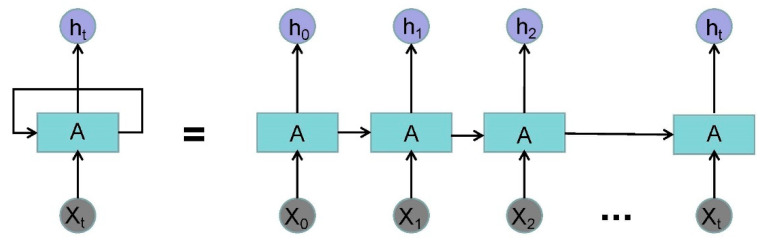
The expansion structure of recurrent neural networks (RNN). (Where $X_{t}$ is the input data of time t and $h_{t}$ is the output data of time t.)

A 3-layer RNN was designed to extract the time feature from the original waveform [32]. For RNN, a very important concept is time. RNN gives an output to the input at each time in conjunction with the state of the current model. From the expansion structure of RNN (Figure 5), it can solve the problem efficiently, which is related to time series.

### 4.2. Multi-Convolutional Neural Network (MCNN)

**Figure 6:**
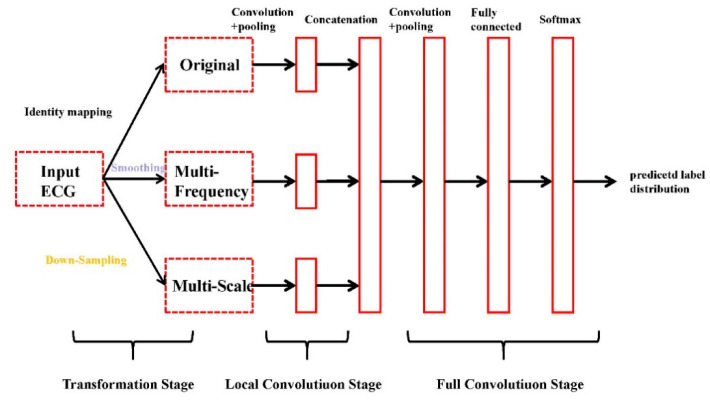
The overall architecture of multi-convolutional neural network (MCNN) [34].

Instant heart rate sequence is extracted from the ECG signal, then an end-to-end multi-scale convolution neural network (MCNN) uses the instantaneous heart rate sequence as the input and the detection result as the output to detect AF [33]. MCNN automatically extracts features at different locations and scales, which makes the model obtain better accuracy in time series data. The overall architecture of MCNN is shown in Figure 6.

The architecture of the MCNN is shown in Figure 6. As shown in the figure, the MCNN framework has three sequential stages: the transformation stage, the local convolution stage, and the full convolution stage.

The MCNN detects AF with the instant heart rate sequence (*IHR*) as input. First, the R location is read from the corresponding annotations. Determine RR intervals according to the R position. Then, *IHR* is calculated by:

(2) $$IHR_{i}=60*\frac{f}{RRI_{i}}$$

where $\;IHR_{i}$ is the ith IHR; f is the sample rate of ECG signal; and $RRI_{i}$ is the ith RR interval.

Afterward, considering that 128 beats are required for the detection of AF, we took 63 IHRs forward and backward for each *IHR*.

### 4.3. 8-layer CNN with Shortcut Connection and 1-layer LSTM (8CSL)

A combination of the 8-layer CNN with shortcut connection and 1-layer LSTM was developed for the ECG classification task. The model was named 8CSL, and includes eight shortcut connections to improve the data transmission and processing speed of traditional CNN.

When training, the data are sent in convolutional neural networks in batches. This article modified the network while ensuring network convergence and improving the generalization ability of the network, which is why the model uses batch-normalization [35]. Rectified linear activation (ReLU) units are introduced as a model requiring a non-linear relationship [36]. Due to the definition of ReLU, when the input is positive, the problem of gradient saturation can be better avoided. The ReLU function is shown in Equation (3).

(3) $$f\left(x\right)=\{\begin{array}{cc} 0, & x<0\\ x, & x\geq 0 \end{array}$$

**Figure 7:**
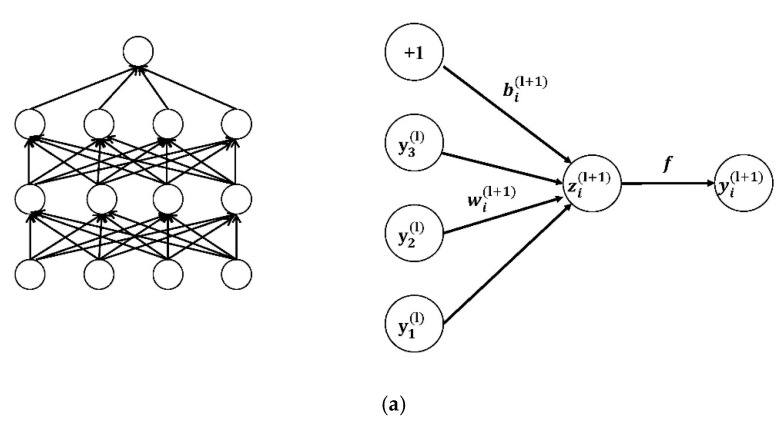

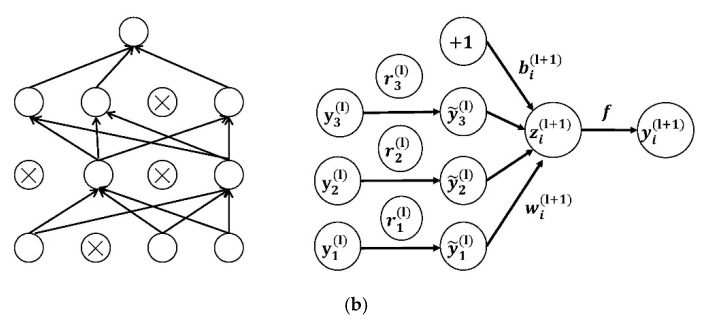
(**a**) The networks not using dropout; (**b**) the networks using dropout [38].

Dropout is then used to reduce the over-fitting of CNN on the training data after the convolution layer [37]. Figure 7 presents the architecture of the dropout.

Equations (4) and (5) are the network calculations without the dropout layer:

(4) $$z_{i}^{\left(l+1\right)}=w_{i}^{\left(l+1\right)}y^{l}+b_{i}^{\left(l+1\right)}$$

(5) $$y_{i}^{\left(l+1\right)}=f\left(z_{i}^{\left(l+1\right)}\right)$$

where $z_{i}^{\left(l+1\right)}$ represents the weighted sum of the input of the ith unit in the (l+1)th layer; weight $w_{i}^{\left(l+1\right)}$ is the (l+1)th neuron; $y^{l}$ is the neuron of lth; $b_{i}^{\left(l+1\right)}$ is the bias of the ith unit in the (l+1)th layer; f is the activation function; and $y_{i}^{\left(l+1\right)}$ represents the weighted sum of the input of the ith unit in the (l+1)th layer.

Equations (6)–(9) are the network calculations with the dropout layer:

(6) $$r_{i}^{\left(l\right)}=Bernoulli\left(p\right)$$

(7) $${\tilde{y}}^{\left(l\right)}=r^{\left(l\right)}\times y^{\left(l\right)}$$

(8) $$z_{i}^{\left(l+1\right)}=w_{i}^{\left(l+1\right)}{\tilde{y}}^{\left(l\right)}+b_{i}^{\left(l+1\right)}$$

(9) $$y_{i}^{\left(l+1\right)}=f\left(z_{i}^{\left(l+1\right)}\right)$$

where r is the mask vector randomly generated by the Bernoulli probability distribution (0–1); the vector element is 0 or 1; the probability of 1 is p; and the probability of 0 is 1−p. In the dropout layer, the r vector is multiplied by the corresponding element of the neuron. If the element in r is 1, it is reserved; if it is 0, it is set to 0. Then only the corresponding parameters of the reserved neuron are trained.

The convolution layer is an important component of the learning features of CNN including a 10 × 1 filter to extract features from the data. When AF features are detected, the features are marked by convolution kernels.

To reduce the risk of over-fitting and the calculation of parameters, the average pool layer and max pool layer are used. Primarily, in this paper, the max pool layer was used as a shortcut connection, which processes a part of the transmitted data.

**Figure 8:**
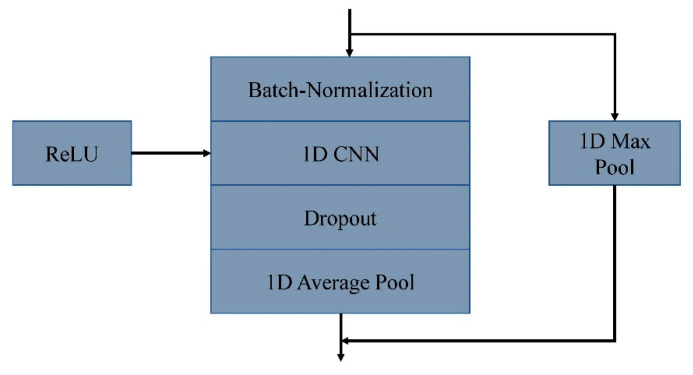
A convolution block based on shortcut connections.

In 8CSL, a convolution block based on shortcut connections is composed of batch-normalization, 1D CNN, ReLU, Dropout, 1D Average pool, and 1D Max pool, as shown in Figure 8.

The feature extracted by the convolutional neural network was input into LSTM to process the feature data. LSTM was added to address the long-term dependency of the data.

The reason why LSTM can better handle the long-term dependency of data is that it relies on the internal memory cell $c_{n}$, which is controlled by various gates to add or delete information.

Equations (10) and (11) are used to describe the information update of the memory cell.

(10) $$c_{n}=f_{n}c_{n-1}+i_{n}{\tilde{c}}_{n}$$

(11) $${\tilde{c}}_{n}=\tan h\left(b_{c}+U_{c}^{T}X_{n}+W_{c}h_{n-1}\right)$$

where f is the forget gate; i is the input gate; ${\tilde{c}}_{n}$ is the newly added information; b is the bias; U is the vector corresponding to the input gate; $X_{n}$ is the data of the input sequence at time n; and $h_{n-1}$ is the hidden layer information at time *n*−1.

To generate a result, the data processed by LSTM were converted into vector values of 2 × 1 using a full connection layer, corresponding to each class (N, A). A Softmax function is used to represent these values as a probability by normalizing them between 0 and 1.

To verify the effect of length factors on performance, the model took 5, 10, and 20 s long segments as input. The output of the model was the probability of each class, and the predictive class of the experimental results is the class with the maximum probability. To reduce memory requirements and better tune parameters, the Adam optimizer was used in the model.

**Figure 9:**
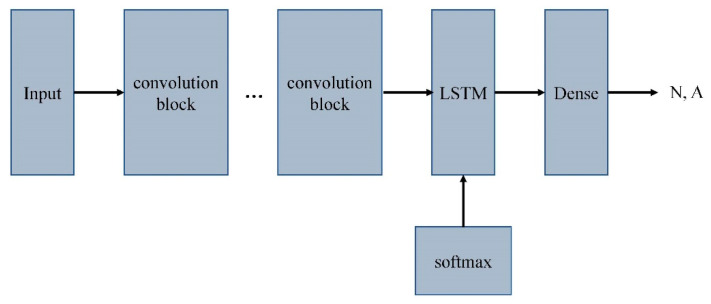
The overall architecture of 8CSL.

The overall architecture of 8CSL is shown in Figure 9.

## 5. Classification Performance Evaluation Index

In this paper, the experiment was carried out in the Keras framework of the Windows 7 operating system. Three models were evaluated based on the test set of the Computing in Cardiology Challenge 2017 dataset with sensitivity (Sen), specificity (Spe), precision (Pre), accuracy (Acc), and F1 score. The experiments were divided into three groups, and the variables were the length of the experimental data.

To calculate the evaluation indexes, true positive (TP), true negative (TN), false positive (FP), and false negative (FN) were adopted, and the calculation formulae are as follows:

(12) $$Sen=\frac{TP}{TP+FN}$$

(13) $$Spe=\frac{TN}{TN+FP}$$

(14) $$Pre=\frac{TP}{TP+FP}$$

(15) $$Acc=\frac{TP+TN}{TP+TN+FP+FN}$$

(16) $$F1=\frac{2\times Sen\times Pre}{Sen+Pre}$$

At the same time, the loss and accuracy curves of the three deep learning models were also calculated. Then, the experimental results with the experimental data of 5 s, 10 s, and 20 s are described, respectively.

In this paper, we used categorical_crossentropy loss as the loss function of the model, which is used to evaluate the difference between the probability distribution obtained from the current training and the true distribution.

The loss is derived from Equation (17):

(17) $$L=-\frac{1}{n}\underset{x}{\sum }\left[y\ln a+\left(1-y\right)\ln\left(1-a\right)\right]$$

where y is the desired output and a is the true output.

## 6. Results

### 6.1. Experiments of 5 Second Segment

**Figure 10:**
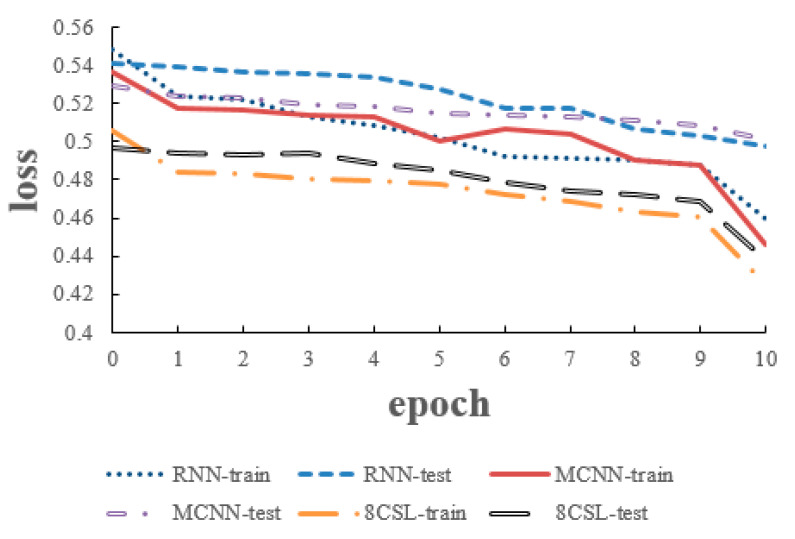
Loss curves of the three models on the five second data.

**Figure 11:**
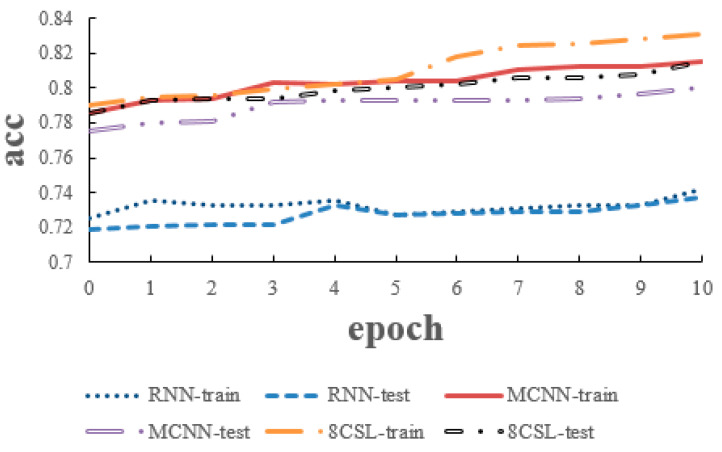
Accuracy curves of the three models on the five second data.

The loss and accuracy curves of the three models are shown in Figure 10 and Figure 11 when the segment length is 5 s.

As can be seen from the Figure 10 and Figure 11, in 8CSL, the minimum loss was 0.4254, and the maximum accuracy was 83.06% on the training set with the minimum loss of 0.4382 and maximum accuracy of 81.53% on the test set. Compared with the other models, 8CSL had the highest accuracy and the lowest loss value, according to the stability and minimum value of the loss curve change and the maximum value of an accuracy curve to judge the best performance of 8CSL.

### 6.2. Experiments of 10 Second Segment

**Figure 12:**
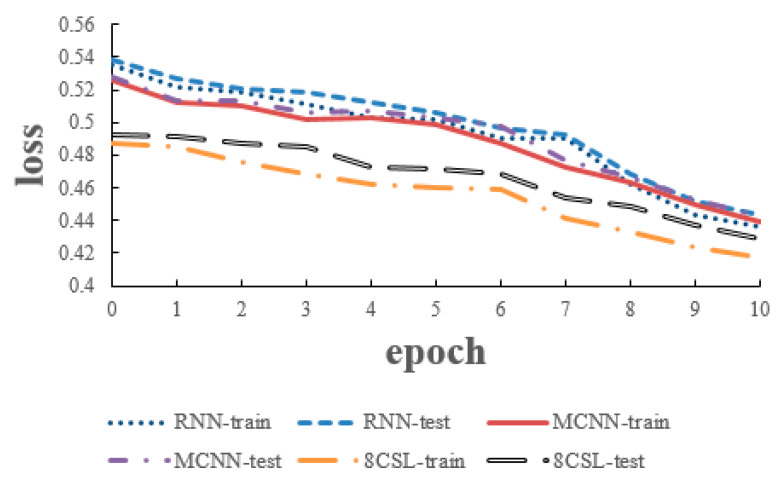
Loss curves of the three models on the 10 second data.

**Figure 13:**
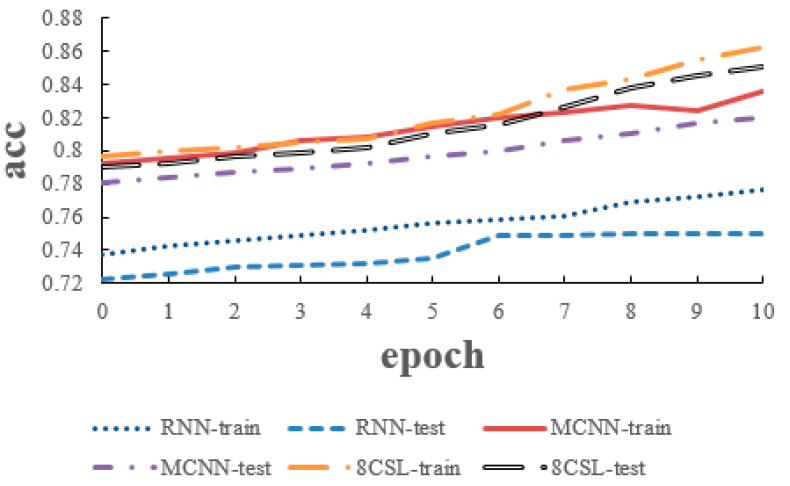
Accuracy curves of the three models on the 10 second data.

The loss and accuracy curves of the three models are shown in Figure 12 and Figure 13, when the segment length was 10 s.

As can be seen from Figure 12 and Figure 13, in 8CSL, the minimum loss was 0.4168, and the maximum accuracy was 86.23% on the training set with the minimum loss of 0.4285 and maximum accuracy of 85.06% on the test set. 

### 6.3. Experiments of 20 Second Segment

**Figure 14:**
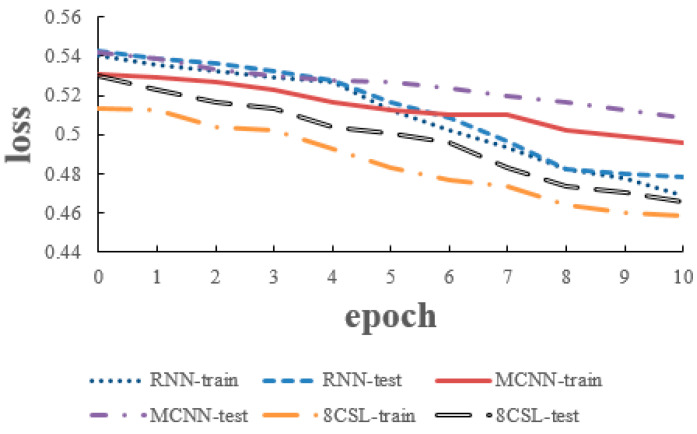
Loss curves of the three models on the 20 second data.

**Figure 15:**
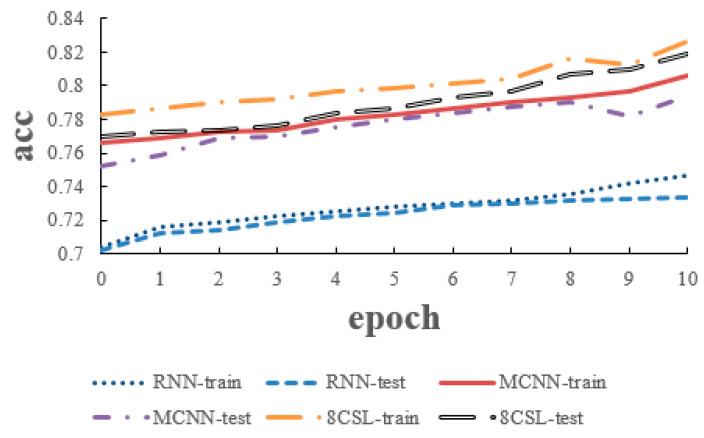
Accuracy curves of the three models on the 20 second data.

The loss and accuracy curves of the three models are shown in Figure 14 and Figure 15 when the segment length was 20 s.

As can be seen from Figure 14 and Figure 15, in 8CSL, the minimum loss was 0.4168, the maximum accuracy was 86.23% on the training set with the minimum loss of 0.4285 and maximum accuracy of 85.06% on the test set.

### 6.4. Overall Results

Table 4 is derived from Equations (12)–(16).

The overall experiments of the three models are shown in Table 4.

As shown in Table 4, in 8CSL, when the segment length is 5 s, the Sen, Spe, Pre, Acc, and F1 score are 84.36%, 89.26%, 85.43%, 81.53%, and 84.89% respectively. When the segment length is 10 s, the Sen, Spe, Pre, Acc, and F1 score are 87.42%, 91.37%, 91.78%, 85.06%, and 89.55% respectively. When the segment length is 10 s, the Sen, Spe, Pre, Acc, and F1 score are 83.08%, 87.21%, 88.37%, 81.86%, and 85.64% respectively. Compared with other models, 8CSL has the best performance on the test set. Because 8CSL has a deeper network model and more ways to prevent over-fitting.

And compared with the same model, the three models all perform best in the segment length is 10 s. Because most of the processed data is distributed around 10 s, the information is relatively complete.

### 6.5. Efficiency Experiment of Shortcut Connection

To validate the effectiveness of shortcut connection in speeding up the data processing, this paper compares the average time of 10 epochs in the case of adding the 8 shortcut connections and no shortcut connection, as shown in Table 5.

As shown in Table 5, if the model use shortcut connection, the data processing time will be saved by 7 s, i.e., the data transmission and data processing are speeded up by 38%.

To verify whether the number of shortcut connections affects the experimental results, we use 6 shortcut connections, 7 shortcut connections, 8 shortcut connections, 9 shortcut connections and 10 shortcut connections to compare.

**Figure 16:**
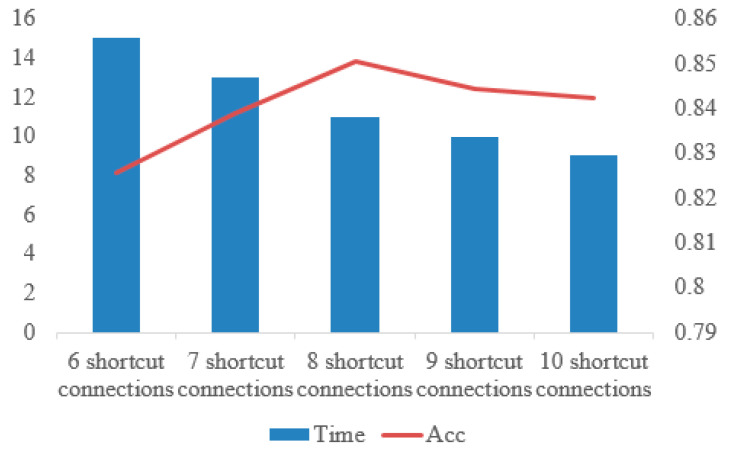
Comparison of time and accuracy under different numbers of shortcut connections.

As shown in Figure 16, the time decreases with the increase of the number of shortcut connections, but the accuracy reaches the highest when the number of shortcut connections is 8. In this paper, on the premise of ensuring the accuracy and time, we choose 8 shortcut connections.

## 7. Conclusions

In this paper, we proposed a combination of an 8-layer CNN with eight shortcut connections and a 1-layer LSTM model, which was to detect AF from single lead short ECG recordings. This can speed up the data transmission and processing of traditional convolutional neural networks by adding shortcut connections. It also consists of a 1-layer LSTM and a fully connected layer, which can not only extract features skillfully, but also deal with long-term dependence between data. The three deep learning models were evaluated based on the test set of the Computing in Cardiology Challenge 2017 dataset with an F1 score. Through three groups of comparative experiments, performance on all indexes of 8CSL was better than that of RNN and MCNN. At the same time, the effectiveness of adding shortcut connections was verified through an efficiency experiment of the shortcut connection. Moreover, 8CSL can be improved by detecting atrial fibrillation in a 12-lead ECG. This direction is the focus of our future research.

## Figures and Tables

**Table 1:** Models and results in references.

Reference	Model	Result			
		Acc	Sen	Spe	F1
[8]	CNN	81.5%	/	/	/
[9]	DCNN	/	/	/	80%
[10]	DCNN	/	/	/	/
[11]	CNN	97.89%	97.12%	96.99%	/
[12]	DCNN	/	/	/	/
[13]	CNN	82%	/	/	/
[14]	CNN and IENN	98.8%	98.6%	/	/
[15]	RNNs	/	/	/	/
[16]	RNN, LSTM, and GRU	95%	/	/	/
[17]	DRNN and LSTM	98.51%	/	/	/
[18]	LSTM	/	/	/	/
[19]	CNN and MENN	97.4%	97.9%	97.1%	/

**Table 2:** The distribution of datasets.

Type	Recording	Time Length (s)				
		Mean	SD	Max	Median	Min
Normal	5154	31.9	10.0	61.0	30	9.0
AF	771	31.6	12.5	60.0	30	10.0
Other rhythms	2557	34.1	11.8	60.9	30	9.1
Noisy	46	27.1	9.0	60	30	10.2

**Table 3:** Accuracy for the different data.

Data	Acc
Normalized Data	85.06%
Original Data	80.23%

**Table 4:** Classification performance of the three models on the test dataset.

Model	Input Length	Sen	Spe	Pre	Acc	F1
RNN	5 s	59.21%	73.31%	59.39%	73.78%	59.30%
	10 s	69.03%	73.19%	59.00%	75.03%	63.62%
	20 s	65.27%	73.13%	58.14%	73.38%	61.50%
MCNN	5 s	81.87%	87.72%	75.50%	80.03%	78.56%
	10 s	82.92%	88.81%	81.94%	82.03%	82.43%
	20 s	80.68%	88.57%	80.31%	79.37%	76.68%
8CSL	5 s	84.36%	89.26%	85.43%	81.53%	84.89%
	10 s	87.42%	91.37%	91.78%	85.06%	89.55%
	20 s	83.08%	87.21%	88.37%	81.86%	85.64%

**Table 5:** Data processing speed with or without shortcut connections.

Model	Speed
Shortcut connection	11 s
No shortcut connection	18 s
